# Atypical TDP‐43 protein expression in an ALS pedigree carrying a p.Y374X truncation mutation in *TARDBP*

**DOI:** 10.1111/bpa.13104

**Published:** 2022-07-24

**Authors:** Johnathan Cooper‐Knock, Thomas H. Julian, Emily Feneberg, J. Robin Highley, Maurice Sidra, Martin R. Turner, Kevin Talbot, Olaf Ansorge, Scott P. Allen, Tobias Moll, Tatyana Shelkovnikova, Lydia Castelli, Guillaume M. Hautbergue, Christopher Hewitt, Janine Kirby, Stephen B. Wharton, Richard J. Mead, Pamela J. Shaw

**Affiliations:** ^1^ Sheffield Institute for Translational Neuroscience (SITraN) University of Sheffield Sheffield UK; ^2^ Nuffield Department of Clinical Neurosciences University of Oxford Oxford UK; ^3^ Neurology Department, Klinikum Rechts der Isar Technical University of Munich Munich Germany; ^4^ Academic Unit of Neuropathology University of Oxford Oxford UK; ^5^ Amarin UK Limited Amarin Corporation London UK

**Keywords:** amyotrophic lateral sclerosis, genetics, neuropathology, protein aggregation, TDP‐43

## Abstract

We describe an autosomal dominant, multi‐generational, amyotrophic lateral sclerosis (ALS) pedigree in which disease co‐segregates with a heterozygous p.Y374X nonsense mutation within TDP‐43. Mislocalization of TDP‐43 and formation of insoluble TDP‐43‐positive neuronal cytoplasmic inclusions is the hallmark pathology in >95% of ALS patients. Neuropathological examination of the single case for which CNS tissue was available indicated typical TDP‐43 pathology within lower motor neurons, but classical TDP‐43‐positive inclusions were absent from motor cortex. The mutated allele is transcribed and translated in patient fibroblasts and motor cortex tissue, but overall TDP‐43 protein expression is reduced compared to wild‐type controls. Despite absence of TDP‐43‐positive inclusions we confirmed deficient TDP‐43 splicing function within motor cortex tissue. Furthermore, urea fractionation and mass spectrometry of motor cortex tissue carrying the mutation revealed atypical TDP‐43 protein species but not typical C‐terminal fragments. We conclude that the p.Y374X mutation underpins a monogenic, fully penetrant form of ALS. Reduced expression of TDP‐43 combined with atypical TDP‐43 protein species and absent C‐terminal fragments extends the molecular phenotypes associated with TDP‐43 mutations and with ALS more broadly. Future work will need to include the findings from this pedigree in dissecting the mechanisms of TDP‐43‐mediated toxicity.

## Graphical abstract

In this study Cooper‐Knock et al identified an autosomal dominant ALS pedigree associated with p.Y374X‐TARDBP. In patient tissue the authors confirmed reduced TDP‐43 protein expression, abnormal TDP‐43 fragmentation and deficient TDP‐43‐mediated splicing. This work extends the phenotypes associated with TDP‐43 mutations.

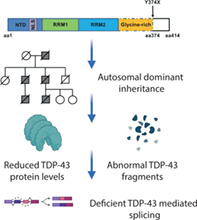

## 1 INTRODUCTION

Amyotrophic lateral sclerosis (ALS) is a fatal and rapidly progressive neurodegenerative disease where death usually occurs within 3–5 years of onset as a result of neuromuscular respiratory failure [1]. Approximately 5%–10% of ALS cases are inherited, and a large number of gene defects have been identified [2]. The neuropathological hallmark of ALS is ubiquitylated neuronal and glial cytoplasmic inclusions containing TDP‐43, the 43kDA protein product of the *TARDBP* gene [3]. This finding, in combination with the identification of dominant mutations in the *TARDBP* gene in familial ALS cases, established a central role for TDP‐43 in the pathophysiology of ALS [4]. Interestingly >95% of ALS cases demonstrate TDP‐43 pathology irrespective of a causal genetic mutation and it has been suggested that the severity of motor neuron degeneration may be proportional to the level of aggregated TDP‐43 [5].

A p.Y374X (subsequently referred to as Y374X) truncating mutation within the TDP‐43 has been described previously in two ALS cases, one of which also carried a p.P525L *FUS* mutation [6, 7]. Neither of the previously reported cases were familial and categorisation of this mutation has been limited to date. Here, we report a multi‐generational ALS pedigree in which disease co‐segregates with a heterozygous Y374X (c.1119_1120delTT) mutation; we performed detailed neuropathological analysis of brain and spinal cord from a single Y374X‐ALS patient. The Y374X mutation occurs within the glycine‐rich, relatively unstructured, C‐terminal domain of the TDP‐43 protein which contains the majority of other reported ALS‐associated mutations. We hypothesised that the Y374X C‐terminal truncation might disrupt the production of C‐terminal fragments which has, up until now, been a prominent hypothesis proposed for TDP‐43‐associated neuronal toxicity [8]. Recently, it was reported that loss of nuclear TDP‐43 function leads to altered splicing of mRNA targets including *UNC13A* and *STMN2*, leading to nonsense‐mediated decay (NMD) and reduced protein expression [9, 10]. Importantly both UNC13A and STMN2 have been linked specifically to neurotoxicity in ALS [11, 12, 13, 14]. Using motor cortex tissue and fibroblasts from Y374X TDP‐43 ALS patients we examined both TDP‐43 fragmentation and TDP‐43 splicing function, including expression of *UNC13A* and *STMN2*. The Y374X TDP‐43 mutation leads to reduced overall protein expression in all tissues examined. Within motor cortex we did not find typical TDP‐43‐positive cytoplasmic inclusions or classical C‐terminal fragmentation, but we did identify atypical TDP‐43 insoluble protein species and evidence of loss of nuclear TDP‐43 splicing function. This case report confirms the pathogenicity of the Y374X TDP‐43 mutation and extends the range of molecular phenotypes associated with TDP‐43 dysfunction in ALS.

## 2 METHODS

### 2.1 Ethics statement

Informed consent was obtained from all participants before skin sample collection (Study number STH16573, Research Ethics Committee reference 12/YH/0330). Autopsy tissues were donated to the Sheffield Brain Tissue Bank (SBTB) with appropriate consent. The SBTB Management Board gave ethical approval for this study under the provision to act as a Research Tissue Bank as approved by the Scotland A Research Ethics Committee (Ref. 08/MRE00/103).

### 2.2 Genetic screening

Polymerase chain reaction (PCR) amplification and direct sequencing of the *TARDBP* gene in kindred members were performed as previously described [15]. In addition, subjects were screened for known ALS genes using a custom designed Agilent SureSelect kit [16].

### 2.3 Fibroblast culture

Fibroblasts were obtained from the Sheffield Motor Neuron Disease Biosample Bank. Control fibroblasts were selected to be age and sex matched with those from the ALS patients.

Fibroblasts were cultured at 37°C, 5% CO_2_ in minimum essential medium (MEM) with Earle's salts and l‐glutamine PAA (Lonza; BE12‐611F) plus 10% foetal calf serum (Biosera), 1X penicillin–streptomycin (Lonza;17‐602E), 1X MEM Vitamins Solution (Lonza; 11120‐037), 1X MEM non‐essential aa (Sigma; S863640C), 1 mM Na pyruvate (Thermo Fisher Scientific; HYC‐001‐196J 40C) and 0.5 mg/ml uridine (Sigma, U‐3003‐5G). Cells were passaged when confluent by dissociation with 1X trypsin (Lonza, BE02‐007E). Cells used for analysis were between passage 9 and 14.

### 2.4 Immunoblotting in fibroblasts

Fibroblasts were dissociated from culture flasks using 1X trypsin and cells collected by centrifugation. The cell pellet was washed once in phosphate‐buffered saline (PBS) and then resuspended in RIPA lysis buffer (Sigma, R0278‐500 ml) plus complete mini EDTA‐free protease inhibitor cocktail (Roche, 11836170001). The lysate was centrifuged at 17,000*g* for 10 min and the supernatant collected. The pellet was solubilised in 7 M urea, 2 M thiourea, 4% CHAPS. Protein concentration was ascertained by Bradford assay. Twenty to thirty micrograms of protein was loaded per lane on a 15% sodium dodecyl sulphate–polyacrylamide gel electrophoresis (SDS‐PAGE) gel with a 4.5% stacking gel and resolved at 100 V for 4 h. Proteins were transferred onto polyvinylidene fluoride membrane (Millipore; IPVH00010) and then blotted with monoclonal mouse anti‐TDP‐43 antibody (Proteintech; 60019‐2; 1:500), which has been epitope mapped to residues 203–209 of human TDP‐43 [17], and mouse anti‐tubulin antibody (Thermo Fisher Scientific; 62204, 1:5000) in 1% cow's milk TBST for 1 h at room temperature (RT). Membranes were washed and then blotted with a secondary antibody, goat anti‐mouse‐HRP (Dako; P0448; 1:5000) for 1 h at RT. Membranes were imaged using EZ‐ECL reagent (Geneflow; 20‐500‐120) on a G‐box (Syngene). Densitometry analysis was performed using G‐box software.

### 2.5 qPCR and splicing assays in fibroblasts

Fibroblasts were dissociated from culture flasks using 1X trypsin and cells collected by centrifugation. The cell pellet was washed once in PBS and then resuspended in RNA‐later (Sigma; R0901‐500 ml) and stored at −20°C. RNA was extracted using RNeasy Mini Kit (Qiagen; 74104) with on‐column DNaseI digest (Qiagen; 79254). RNA was extracted from 60 to 70 mg of fresh frozen frontal cortex using RNeasy lipid tissue mini kit (Qiagen; 74804) with on‐column DNaseI digest. RNA concentration was determined using a Nanodrop 2000 (Thermo Fisher Scientific). RNA (1–2 μg total) was reverse transcribed using High‐Capacity cDNA Reverse Transcription Kit (Applied Biosystems; 4387406). qPCR was performed using Brilliant III Ultra‐Fast SYBR Green QPCR Master Mix (Agilent, 600882) and 1 μM primers on a CFX96 QPCR machine (Bio‐Rad) with a 1:10–1:20 dilution of template and the following cycle lengths: 3 min at 95°C (5 s at 95°C, 10 s at 60°C) × 40 cycles. qPCR data were analysed using Bio‐Rad CFX Manager Software and Microsoft Excel. QPCR primer sequences were: *TARDBP* transcript variant 1—forward primer 5′‐CTTTTAGGAGATCATGGTGTCACA‐3′, Reverse primer 5′‐CCTGTGATGCGTGATGACGAA‐3′; 18S RNA—forward primer 5′‐CTGCGG CTTAATTTGACTCAA CA‐3′, reverse primer 5′‐CAAATCGCTCCACCAACTAAGAA‐3′. For the *POLDIP*/*SKAR* splicing assay, semi‐quantitative PCR was performed on cDNA samples using the primers described by Budini et al. [18] and 5X Firepol Green PCR master mix with the following cycling conditions: 5 min at 95°C (30 s at 95°C, 30s at 55°C, 30 s at 72°C). Thirty cycles of amplification were used for POLDIP3/SKAR and 20 cycles for 18S RNA. Five microliters of PCR product was resolved through a 2.5% agarose gel stained with ethidium bromide and imaged with a Geni UV trans‐illuminator system (Syngene). Densitometry on sub‐saturation exposure images was performed using ImageJ.

### 2.6 Immunocytochemistry

Fibroblasts cultured on 5‐mm diameter glass coverslips were fixed in freshly prepared neutrally buffered 4% paraformaldehyde dissolved in PBS. Coverslips were blocked and permeabilised in 5% bovine serum albumin (Sigma, 05479‐50G) 0.1% Triton X‐100, 0.05% SDS, for 1 h at RT. Coverslips were then incubated overnight at 4°C with primary antibodies, mouse monoclonal anti‐TDP‐43 (Proteintech; 600190‐2; 1:500, epitope mapped to residues 203–209) and rabbit anti‐α‐tubulin (Abcam; AB4074; 1:500) diluted in 1% BSA. Coverslips were then washed in 1X PBS and incubated with secondary antibodies, Donkey anti‐mouse‐Alexa555 (Invitrogen; A31570; 1:1000) and donkey anti‐rabbit‐Alexa488 (Invitrogen; A21206; 1:1000) in 1% BSA, for 1 h at RT. Coverslips were then incubated with Hoechst stain (Sigma; 861405‐100MG; 1:500), washed in 1X PBS and mounted using Fluoromount (Sigma; F4680‐25 ml). Coverslips were imaged using Incell2000 (GE Healthcare) and fluorescence intensity data analysed using Incell Developer Software (GE Healthcare).

### 2.7 Neuropathology

The brain and spinal cord from Patient 194 and the control cases were donated to the SBTB with the consent of the next of kin. One cerebral hemisphere, half the midbrain and brainstem, a portion of the cerebellum, and segments of the spinal cord from cervical, thoracic and lumbar levels were snap‐frozen at autopsy using liquid nitrogen. The remaining central nervous system tissue was formalin‐fixed and tissue blocks from association neocortical regions, motor cortex, hippocampus, basal ganglia, thalamus, midbrain, pons, medulla, cerebellum and spinal cord at cervical, thoracic and lumbar regions were processed to paraffin. In addition to routine neuropathologic examination of haematoxylin and eosin stained tissue, immunohistochemistry was performed with antibodies for p62/sequestosome‐1 (BD Biosciences; 610833; 1:200), TDP‐43 (Proteintech; 10782‐2 AP; 1:2000), S409/410 phosphorylated TDP‐43 (Cosmo; TIP‐PTD‐M01; 1:4000) and CD68 (Dako; M0814; 1:100) using standard protocols in spinal cord and motor cortex.

TDP‐43 protein fragmentation in the urea insoluble protein fraction from motor cortex tissue was quantified by immunoblot and mass spectrometry (MS) as previously described [19]. In brief, commercially synthesised heavy peptides (Thermo Fisher Scientific) were used for the absolute quantification of the endogenous light peptides by parallel reaction monitoring (PRM). For interpretation of the MS‐PRM results absolute abundances of the light peptides (log_10_ ratio [light:heavy peptide]) were compared. The C:N‐terminal peptide ratio was calculated by dividing non‐log absolute abundances of the light peptides. Truncation peptides include one cleavage site independent of the enzyme used for digestion (giving a nonspecific digestion pattern), which suggests that cleavage has occurred endogenously prior to digestion.

### 2.8 Immunoblotting in motor cortex tissue

Motor cortex tissue (~0.1 g) was lysed in 400 μl RNase free RIPA lysis buffer (2% SDS, 50 mM Tris, pH 8.0, 150 mM NaCl, 1% IGEPAL CA‐630 [Sigma], 0.5% sodium deoxycholate, benzonase [250 units in 10 ml buffer], protease inhibitor cocktail [Merck], 2 mM PMSF) using a Precellys evolution homogeniser and 8 × 1.4 mm Zirconium Oxide beads at 5500 rpm for 2 × 30 s, 10‐min incubation on ice and another round of homogenisation. Lysates were collected after centrifugation at 17,000 *g* for 10 min. RNA was extracted from 250 μl lysate, with the remainder used for immunoblotting.

Protein extract concentrations were determined using a Bradford Reagent (Bio‐Rad), resolved by SDS‐PAGE electroblotted onto nitrocellulose membrane and probed using the relevant primary antibody. STMN2 (1:1000 dilution) (Proteintech; 10586‐1‐AP) and TDP‐43 (C terminus) (1:1000 dilution) (Proteintech; 12892‐1‐AP) were detected with horseradish peroxidase (HRP)‐conjugated rabbit secondary antibody (Promega), while α‐tubulin, clone DM1A (1:2000) (Insight Biotechnology Ltd.; Sc32293) and TDP‐43 (1:3000) (Abnova; H00023435‐M01) were detected with HRP‐conjugated mouse secondary antibody (Promega). Immunoblots were visualised using a Licor Odyssey Fc and densitometry analysis performed using Image Studio Lite (Version 5.2.5).

### 2.9 qPCR in motor cortex tissue

RNA was extracted from motor cortex using PureZOL (Bio‐Rad). Briefly, one fifth the volume of chloroform was added and tubes were vigorously shaken for 15 s followed by 10 min incubation at RT and centrifugation at 12,000*g*, 10 min, 4°C. The upper phase was collected and the RNA was precipitated overnight at −20°C with equal volume isopropanol and 1 μl glycogen (5 μg μl^−1^; Ambion) and subsequently pelleted at 17,000*g*, 20 min, 4°C. RNA pellets were washed with 70% DEPC ethanol and resuspended in DEPC water. All RNA samples were treated with DNaseI (Roche) and quantified using a Nanodrop (NanoDropTechnologies). Following quantification, 2 μg RNA was converted to cDNA using BioScript Reverse Transcriptase (Bioline). Human snRNA U1 (Fwd: 5′‐CCATGATCACGAAGGTGGTT‐3′, Rev: 5′‐ATGCAGTCGAGTTTCCCACA‐3′); human STMN2 (Fwd: 5′‐GCGTCTGCACATCCCTACAA‐3′, Rev:5′‐ACAGCATGGACAGCTCCTTC‐3′), and human UNC13A (Fwd: 5′‐AAGCCAAGTTTGATGGTGCC‐3′, Rev: 5′‐CATGAAATCCTGCTCCCAGC‐3′) primers were used in this study. qRT‐PCR reactions were performed in duplicate using the Brilliant III Ultra‐Fast SYBR Green QPCR Master Mix (Agilent Technologies) on a C1000 TouchTM thermos Cycler using the CFX96TM Real‐Time System (Bio‐Rad) using an initial denaturation step, 45 cycles of amplification (95°C for 30 s; 60°C for 30 s; 72°C for 1 min) prior to recording melting curves. qRT‐PCR data were analysed using CFX Manager software (Version 3.1) (Bio‐Rad) and quantification performed using the comparative CT method (35) and GraphPad Prism (Version 9).

## 3 RESULTS

### 3.1 The p.Y374X TDP‐43 mutation co‐segregates with ALS in a multi‐generational pedigree

**FIGURE 1:**
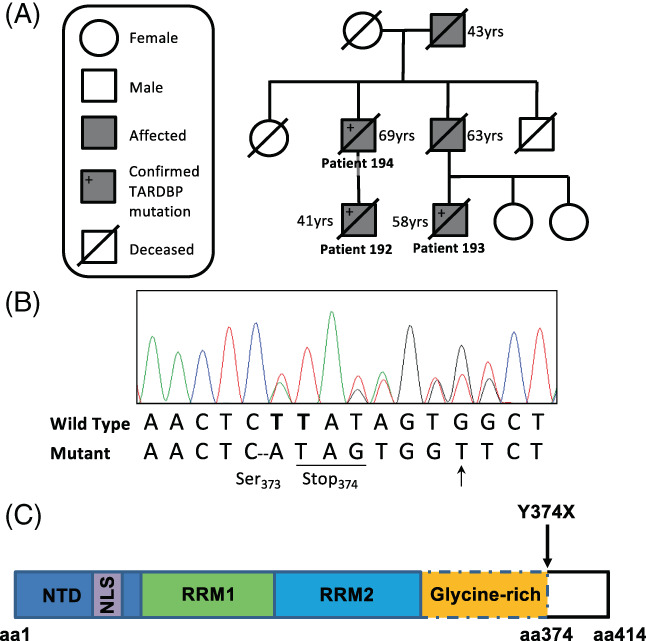
Characterisation of the kindred. (A) Graphical representation of the kindred. (B) Sequencing chromatograph from a patient with the c.1119_1120delTT (Y374X) mutation. Novel synonymous SNP also shown (arrow): C.1128C>T (G376G). (C) Domain structure of the TDP‐43 protein including position of the N‐terminal domain (NTD), nuclear localisation signal (NLS), RNA‐recognition motifs (RRM) and C‐terminal glycine‐rich domain which is truncated by the Y374X mutation

A Y374X truncating mutation in TDP‐43 (c.1119_1120delTT) was identified in the present study. This mutation was detected in three affected kindred members (Figure 1A,B). In addition, a novel synonymous SNP, c.1128C>T (p.G376G) was also identified in the kindred (Figure 1B). The kindred was negative for known ALS‐associated mutations [20] (Table S1). Y374X TDP‐43 is absent from 141,456 population controls in gnomAD v2.1.1 [21]; it is also predicted to be pathogenic (CADD > 20) [22]. In addition Y374X TDP‐43 has been previously identified in ALS patients [6, 7] although co‐segregation with familial ALS has not been previously demonstrated. The synonymous p.G376G change is also rare (allele frequency 0.000023 in gnomAD v2.1.1) but it is not predicted to be pathogenic, CADD = 8, reported ‘likely benign’ in ClinVar [23]. Overall, we determined that the Y374X truncation is the likely cause of ALS in this pedigree.

### 3.2 Clinical features

The index pedigree is shown in Figure 1A. The index case (Patient 192) developed limb weakness aged 39 years. On examination he was found to have a pure motor syndrome with mixed upper and lower motor neuron features and was subsequently diagnosed with ALS following detailed neurological investigation. He progressed to develop respiratory involvement requiring non‐invasive ventilation 2 years after symptom onset. He was unfortunately killed in a road traffic accident 25 months after symptom onset. His father (Patient 194) presented with limb weakness aged 67 years. He also displayed signs of mixed upper and lower motor neuron dysfunction and followed a similar disease course requiring non‐invasive ventilation, but not gastrostomy, before dying from respiratory failure 29 months after symptom onset. The third patient in the kindred (Patient 193) was the cousin of the index case. He developed limb weakness at the age of 53 years. His disease course was longer than the other two family members, dying from respiratory failure 5 years after symptom onset. He required both non‐invasive ventilation and gastrostomy insertion prior to his death. The grandfather and the paternal uncle of the index case also suffered from ALS, however, genetic material was not available from either of these individuals. Both had limb‐onset disease, the grandfather dying aged 43 years and the uncle aged 63 years.

### 3.3 Neuropathology

**FIGURE 2:**
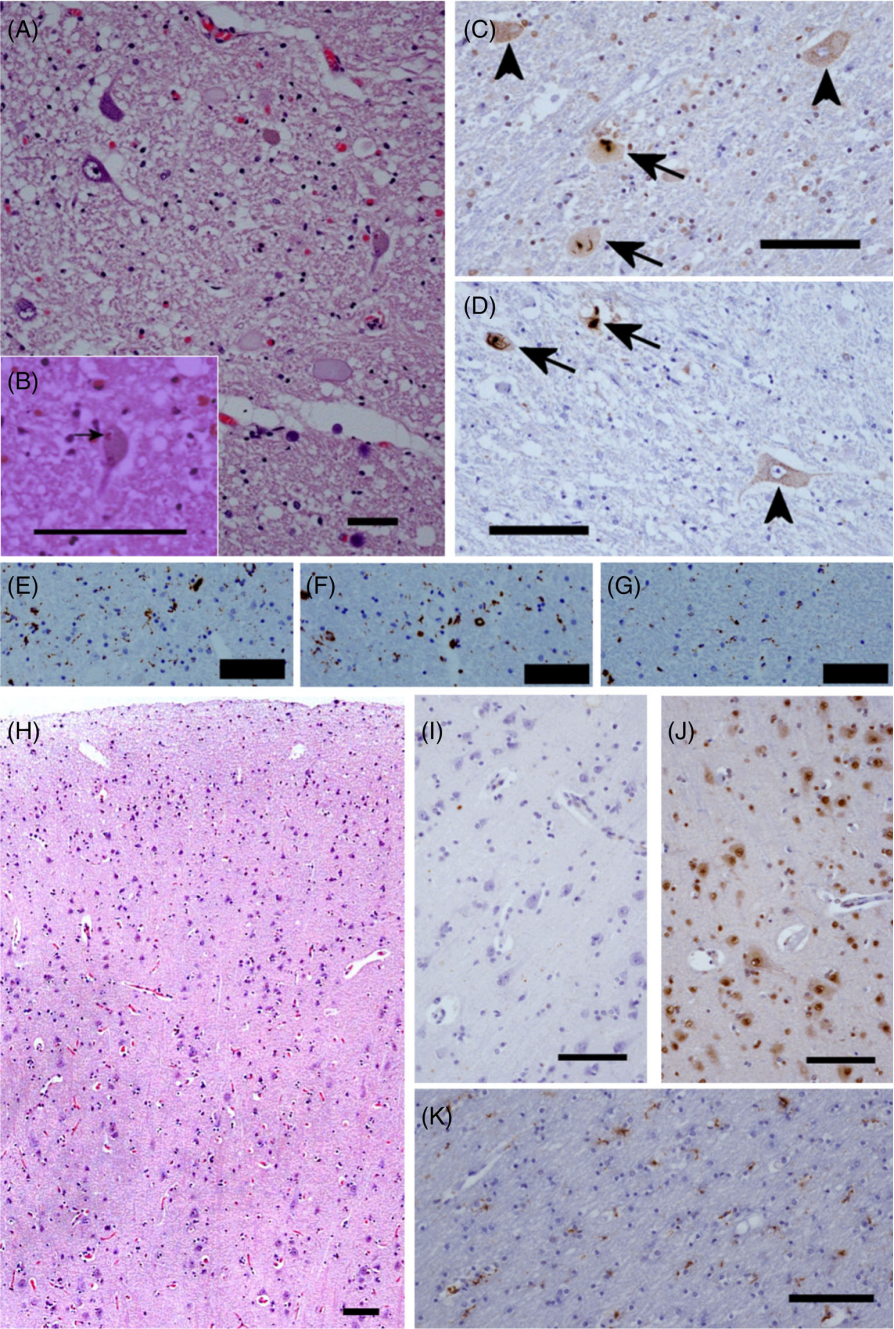
Neuropathology of a patient with Y374X mutation. Microscopic examination of the spinal cord reveals: Motor neuron loss (A) with Bunina bodies in occasional residual neurons (arrow, B, H&E); neuronal cytoplasmic inclusions (arrows) and pre‐inclusions (arrow heads) (C and D) on immunohistochemistry for total TDP‐43 (C) and phosphorylated TDP‐43 (D); a microglial reaction (anti‐CD68) in the anterior horns (E) and lateral columns (F), that is not seen in the dorsal columns (G). Examination of the motor cortex is essentially normal on H&E (H) with minimal microglial reaction on CD68 immunohistochemistry (K) and an absence of glial or neuronal cytoplasmic inclusions on immunohistochemistry for TDP‐43 (J) or p62 (I); bar = 100 μm throughout

Neuropathological features were most prominent in lower motor neurons. There was loss of lower motor neurons that was most marked in the anterior horns of the spinal cord (Figure 2A) with Bunina bodies in residual neurons (Figure 2B). Immunohistochemistry revealed a number of neuronal cytoplasmic inclusions (both skein‐like and compact) and pre‐inclusions that immuno‐labelled with antibodies to p62, TDP‐43 and S409/410 phosphorylated TDP‐43 (Figure 2C,D). Glial cytoplasmic inclusions were also present in low numbers. Antibodies against CD68 revealed a microglial reaction throughout the spinal cord grey matter (Figure 2E) as well as white matter (including the anterior and lateral descending tracts, Figure 2F) that was less prominent in the dorsal columns (Figure 2G). This microglial reaction was also evident to a lesser extent in the white matter underlying the motor cortex, which showed no significant pathology in the form of neuronal loss, microvacuolation, neuritic pathology, neuronal or glial cytoplasmic or intranuclear inclusions on haematoxylin and eosin staining or on immunohistochemistry for p62, TDP‐43 or phosphoTDP‐43 (Figure 2H–K). In fact TDP‐43 pathology was confined to anterior horns of the spinal cord and motor neurons within the medulla (including the pyramids, inferior olives, hypoglossal nuclei and reticular formation). No TDP‐43 pathology, including nuclear TDP‐43 mislocalization, was evident on immunohistochemistry within the motor cortex, superior and middle temporal gyri, middle frontal gyrus or posterior hippocampus. Extrapyramidal regions (including the cerebellum, hippocampus and neocortices of the anterior frontal and temporal lobes) did not show stellate, neuronal cytoplasmic inclusions of the type classically associated with mutations of *C9ORF72* [24].

### 3.4 Characterisation of TDP‐43 protein and mRNA expression in fibroblasts and CNS tissue

**FIGURE 3:**
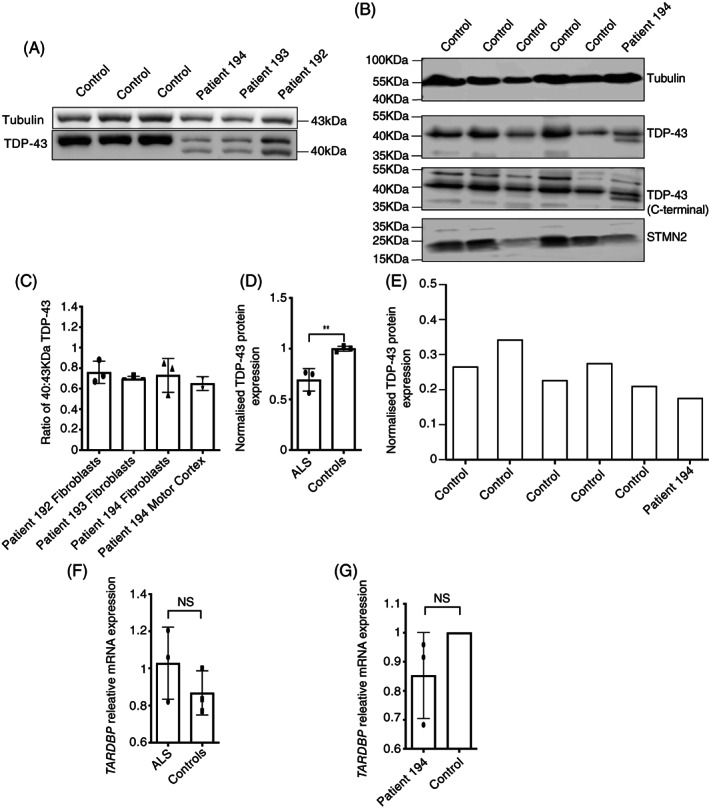
Expression of the truncated protein and *TARDBP* mRNA expression. (A and B) Western blot of protein isolated from patient and control fibroblasts (A) and motor cortex tissue (B). Protein isolated from patients shows an additional 40‐kDa band, which is the predicted molecular weight of the truncated protein, not present in control lysates. The Western blot also includes STMN2 protein which was quantified in parallel. (C) Ratio of 40 kDa to 43 kDa band in fibroblasts and motor cortex tissue lysates; mean and SD from three independent experiments shown for fibroblasts. Results from motor cortex tissue are two independent measurements of a single case. (D and E) Quantification of total TDP‐43 protein, normalised to α‐tubulin, in both fibroblasts (D) and motor cortex tissue (E) obtained from patients with Y374X mutation, compared to controls. For fibroblasts, mean and SD of three patients and three controls, each tested in three independent experiments; unpaired *t*‐test *p* = 0.0096. Results from motor cortex tissue are from a single case and five independent controls. (F and G) Level of *TARDBP* transcript variant 1 mRNA, normalised to 18S RNA and expressed relative to control, in fibroblasts (F) and motor cortex tissue (G) obtained from patients with Y374X mutation and controls. For fibroblasts, mean and SD of three patients and three controls, analysed in three independent experiments shown; unpaired *t*‐test *p* = 0.5490. Results from motor cortex tissue are the mean of a single case–control pairing analysed in three independent experiments

Y374X‐TDP‐43 encodes a truncated form of TDP‐43 including aa 1–374 but predicted to be missing the final 40 C‐terminal aa (Figure 1C **)**. To confirm expression of the mutated allele we analysed protein lysates from cultured fibroblasts, isolated from three members of the kindred, and motor cortex tissue, isolated from one member. The truncated protein was expressed (Figure 3A,B) at the expected molecular weight. Densitometry analysis showed that the concentration of truncated protein was reduced to 65%–76% of the level of the full‐length protein (Figure 3C). Total TDP‐43 protein levels, determined by combining the intensities of both full‐length and truncated protein bands, were also reduced to 61%–69% of controls in both fibroblasts and motor cortex tissue (Figure 3D,E). In our analysis of motor cortex, five normal controls were used for comparison to determine whether TDP‐43 protein expression in the Y374X‐TDP‐43 case is outside the range of values found in normal individuals (Section 2).

We also conducted qPCR analysis to determine whether reduced protein expression was due to transcriptional effects. The levels of *TARDBP* transcript variant 1 mRNA in fibroblasts and motor cortex tissue, normalised to 18S RNA levels, showed no difference between patients with the Y374X mutation and controls (Figure 3F,G). It should be noted that this assay does not distinguish mRNA transcribed from wild‐type and truncated alleles.

### 3.5 Quantification of insoluble TDP‐43 peptide species within CNS tissue

Enrichment of insoluble TDP‐43 is associated with a characteristic immunohistochemical signature including phosphorylated, ubiquitinated and N‐terminal truncated TDP‐43 resulting in C‐terminal fragment enrichment (CTF) [3]. The presence of TDP‐43 CTF has been proposed to contribute to neuronal toxicity [25]. Y374X‐TDP‐43 is a C‐terminal truncating mutation and we therefore investigated whether this would affect C‐terminal fragment formation. We have recently published an extensive characterisation of insoluble TDP‐43 species within CNS tissue including immunoblotting and targeted proteomics (MS‐PRM) enabling antibody‐free quantification of pathological TDP‐43 species [19].

**FIGURE 4:**
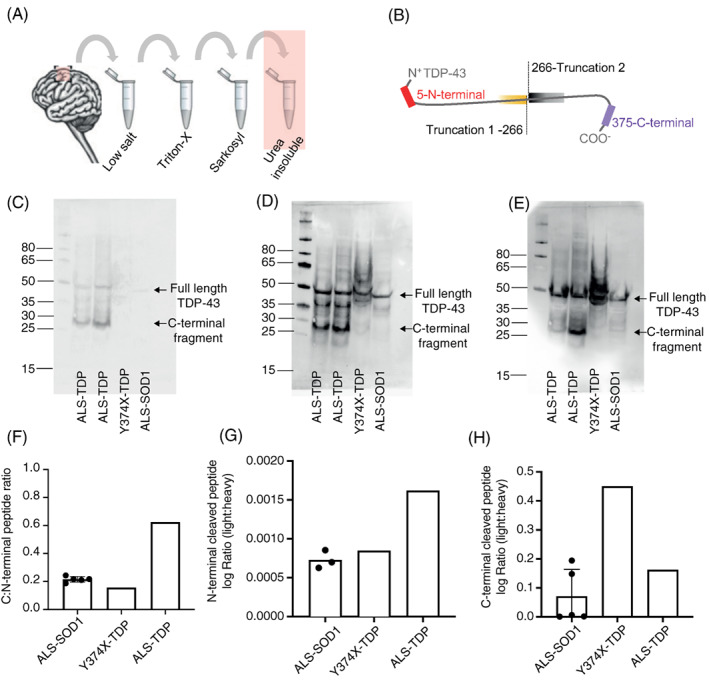
Quantification of insoluble TDP‐43 peptide species within CNS tissue (A) serial fractionation of motor cortex brain tissue for the enrichment of pathological TDP‐43 in the insoluble (urea) protein fraction. (B) The relative abundance of endogenous C‐ and N‐terminal TDP‐43 fragments was measured by MS‐PRM using fixed concentrations of commercially synthesised heavy isotope‐labelled peptides corresponding to the N‐ and C‐terminal ends of the TDP‐43 protein. (C) Immunoblot with an antibody against 409/410 phosphorylated TDP‐43 (Proteintech; 22309‐1) shows phosphorylated full‐length TDP‐43 and C‐terminal fragments in sporadic ALS. (D) The antibody against C‐terminal TDP‐43 (260–274, Proteintech; 12892‐1) reacts with full‐length TDP‐43 and C‐terminal fragments in sporadic ALS. No C‐terminal fragments are present in Y374X and a control ALS case without TDP‐43 pathology. (E) The antibody against N‐terminal TDP‐43 (1‐260 Proteintech; 10782‐2‐AP) labels proteins specific to the Y374X case including a lower molecular TDP‐43 protein at approximately 40 kDa consistent with the C‐terminal Y374X truncation. (F) The C‐ to N‐terminal peptide ratio quantified by MS‐PRM is increased in sporadic ALS but normal in the Y374X case. (G) Quantification of the N‐terminal cleaved peptide reveals an increase in C‐terminal fragments (CTFs) in sporadic ALS. (H) Quantification of the C‐terminal cleaved peptide reveals an increase in N‐terminal protein fragment species in the Y374X case

Motor cortex tissue from a single Y374X‐TDP‐43 ALS patient was fractionated to enrich for pathological insoluble species (Figure 4A) although this tissue did not contain TDP‐43 pathology by immunohistochemistry (Figure 2H–K). The immunoblot pattern associated with the Y374X mutation differed from sporadic ALS, SOD1‐ALS which is not associated with TDP‐43 pathology [26], and control tissue (Figure 4C–E). C‐terminal fragments were not demonstrated in tissue from the Y374X TDP‐43 patient or a p.I113T‐SOD1 ALS patient using an antibody directed against phosphorylated serin at 409/410 located at the C‐terminal end of TDP‐43 (Figure 4C) or an antibody directed against the C‐terminal (Figure 4D) or the N‐terminal (Figure 4E) end of TDP‐43. Immunoblot for the C‐terminal and N‐terminal of TDP‐43 in Y374X demonstrate the presence of full‐length TDP‐43 (43 kDa) and a lower TDP‐43 band at approximately 40 kDa (Figure 4D,E). It should be noted that the C‐terminal antibody used in this analysis binds an epitope including aa 260–274 and would therefore be expected to detect the Y374X truncated protein. We conclude that the 40 kDa band is likely to represent the Y374X N‐terminal protein fragment. A number of additional bands were present at higher molecular weight which are specific to Y374X TDP‐43 (Figure 4D,E); this may be consistent with differential or post‐translational processing of the Y374X TDP‐43 protein.

MS‐PRM enabled antibody‐free quantification of TDP‐43 fragments and confirmed the findings of the immunoblot analysis. Heavy isotope‐labelled peptides corresponding to the N‐ and C‐terminal ends of the TDP‐43 protein (Figure 4B) were synthesised and injected at fixed concentration to enable quantification of the endogenous N‐ and C‐terminal fragments. In Figure 4B, ‘Truncation 1’ corresponds to the isolated N‐terminal of TDP‐43 without the C‐terminal; ‘Truncation 2’ corresponds to the isolated C‐terminal of TDP‐43 without the N‐terminal. In sporadic ALS the C:N‐terminal peptide ratio (Figure 4F) and the N‐terminal cleaved peptide (Truncation 2, Figure 4G) were increased in the insoluble fraction in line with the presence of C‐terminal fragments, compared to ALS caused by p.I114‐T, p.D102N or p.I113T mutations within SOD1. However, in Y374X TDP‐43 the C:N‐terminal ratio was normal (Figure 4F) and there was a relative increase in abundance of the C‐terminal cleaved peptide (Truncation 1, Figure 4H) consistent with active expression of additional N‐terminal protein fragments including but not limited to the C‐terminal truncated Y374X TDP‐43 protein. In Figure 4G, Truncation 2 was undetectable in two measurements of ALS‐SOD1 so these results were not plotted.

### 3.6 Effect of the truncated protein on mRNA splicing

**FIGURE 5:**
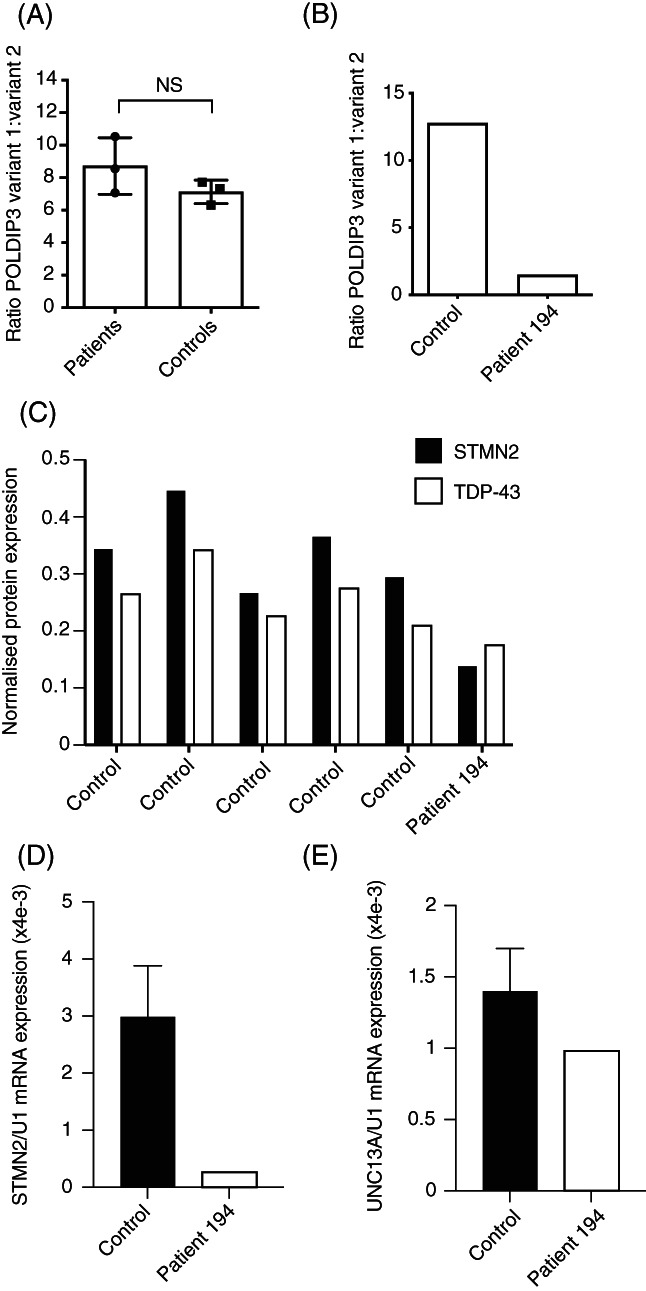
Loss of TDP‐43 splicing activity in the presence of the Y374X‐TDP‐43 mutation. (A and B) Semi‐quantitative PCR revealed no evidence of abnormal *POLDIP3*/*SKAR* gene splicing in fibroblasts (A) from Y374X‐TDP‐43 ALS patients compared to controls; but in motor cortex (B) there was evidence of loss of TDP‐43 function leading to an excess of transcript variant 2. (C and D) Quantification of STMN2 expression in motor cortex tissue from a single patient with a Y374X mutation and five controls. Quantification included STMN2 protein normalised to α‐tubulin (C) and *STMN2* mRNA (D) normalised to U1 snRNA. Across all measurements expression of STMN2 protein was correlated with expression of TDP‐43 (Pearson test, *r* ^2^ = 0.9, *p* = 0.004). (E) Quantification of UNC13A mRNA expression normalised to U1 snRNA in motor cortex tissue from a single patient with a Y374X mutation and five controls

Our analysis suggests that the quantity of total TDP‐43 protein is reduced in the presence of the Y374X mutation (Figure 3D,E) and therefore we set out to measure whether there was a reduction in TDP‐43 function. A semi‐quantitative PCR was performed to determine if splicing activity was altered. Splicing of the *POLDIP3*/*SKAR* gene is altered by loss of TDP‐43 function resulting in exclusion of exon 3, and so RT‐PCR for this gene was performed [18, 27]. The relative quantities of transcript variant 1 and variant 2 (no exon 3) in three patients and three control fibroblast lines were determined by densitometry following agarose gel electrophoresis of RT‐PCR products and were not significantly different (unpaired *t*‐test *p* = 0.2165, Figure 5A). However, the splicing ratio in motor cortex tissue from a single case–control pairing was also determined and indicated increased relative abundance of variant 2 (lacking exon 3) (Figure 5B), as would be expected for a loss of function in TDP‐43 [18].

We also utilised an indirect measure of TDP‐43 splicing function which has been linked directly to ALS pathogenesis. Recently, reports have connected reduced nuclear TDP‐43 content to missplicing of STMN2 and UNC13A and neurotoxicity in ALS [9, 10]. Misspliced STMN2 and UNC13A mRNA includes aberrant cryptic exons which induce nonsense‐mediated mRNA decay (NMD) and consequent reduced protein expression. We hypothesised that reduced expression of TDP‐43 within the motor cortex of the Y374X ALS patient might replicate these defects despite the absence of classical TDP‐43‐positive cytoplasmic inclusions. In line with this hypothesis we identified reduced expression of STMN2 mRNA (91% reduction, Figure 5D **)** and protein (60% reduction, Figure 5C). UNC13A mRNA expression was also reduced (30% reduction, Figure 5E). Despite use of five control individuals to generate these data, statistical interpretation was not possible because of comparison to a single Y374X ALS patient. Consistent with previous observations [9, 10], expression of STMN2 and TDP‐43 protein was tightly correlated across all samples (Pearson test, *r* ^2^ = 0.9, *p* = 0.004).

## 4 DISCUSSION

Here, we have presented data from a multi‐generational ALS kindred with a Y374X truncating mutation within TDP‐43. This is the first time this atypical mutation has been shown to co‐segregate with ALS in a single pedigree, which represents good evidence for monogenic pathogenicity. The age of onset varied significantly in our pedigree which may explain the observation of this mutation previously in an apparently sporadic ALS case [6].

Whilst dysfunction of TDP‐43 is undoubtedly associated with ALS [4] the mechanism of neuronal toxicity is debated. Both loss of function [28] and gain of function [29] mechanisms have been proposed. The present study is limited by small sample size, but available fibroblasts and post mortem tissue enabled us to investigate whether certain molecular observations related to TDP‐43 found in previous studies, were applicable in this pedigree. Where there is deviation from previously reported findings, the inference is that inconsistently observed phenotypes may not be essential for ALS pathogenesis.

The discovery of TDP‐43 CTF [3] was followed by evidence that these fragments were toxic in vitro [25] and could be responsible for motor neuron degeneration. Indeed, a spectrum of insoluble TDP‐43 species has been described correlating with pathological subtype and even disease severity [30]: frontotemporal lobar degeneration or FTLD type TDP‐C [31] is associated with very dense TDP‐43 aggregates as compared to TDP‐43 extracted from FTLD‐TDP‐A or ALS. Our report represents a novel contribution to the understanding of the molecular pathogenesis of TDP‐43‐associated neurodegeneration. We have discovered a distinct TDP‐43 fragmentation pattern, including a number of high molecular weight N‐terminal protein fragment species, which suggests that fragments other than C‐terminal fragments may have a role in the pathophysiology of ALS. Biophysical variability in insoluble TDP‐43 species has been previously described and correlated with cellular toxicity and even disease severity [30]. The present report suggests further diversity in insoluble TDP‐43 species which will inform future translational research.

It should be noted that we tested TDP‐43 fragmentation in motor cortex tissue for consistency with our previous work [19]. However, in this case the motor cortex was devoid of classical TDP‐43 pathology. Analysis of samples from members of the reported kindred showed that the Y374X truncated protein was expressed both in cultured fibroblasts and in central nervous system tissue, and in both the soluble and insoluble fractions. Quantification of the amount of TDP‐43 protein in both fibroblasts and frontal cortex tissue showed that the total amount of TDP‐43 protein was reduced in ALS patients compared to controls. To investigate the function of TDP‐43 we applied direct and indirect measures of TDP‐43 splicing within motor cortex tissue. Crucially, the indirect measure consisted of quantification of STMN2 and UNC13A expression; reduced expression of these proteins is linked to reduced nuclear TDP‐43 function and to the rate of ALS progression [9, 10]. In all measures TDP‐43 function was reduced in the presence of Y374X despite the absence of classical TDP‐43 pathology. We suggest therefore that TDP‐43‐positive cytoplasmic inclusions are not necessary to initiate disease pathophysiology although they may form later as evidenced by the presence of these inclusions within lower motor neurons from our patient.

A caveat in our study of TDP‐43 fragmentation function is that the analysis is based on a single Y374X TDP‐43 ALS patient compared to up to five neurologically normal controls. In addition we focused on the motor cortex which was devoid of classical TDP‐43 pathology in this case. However, we note that there is precedence in the literature for disease‐associated neuronal dysfunction in the absence of classical TDP‐43 pathology. For example, TDP‐43 pathology is relatively more frequent in the spinal cord compared to the motor cortex even when there is clearly clinical evidence of upper motor neuron involvement [32]. In *C9ORF72*‐FTLD neurodegeneration can be associated with RNA foci and dipeptide‐repeat protein inclusions without TDP‐43 pathology [33]. Finally, we provide evidence for atypical TDP‐43 protein species within the motor cortex of our patient which are not present in controls, but clearly it is possible that typical C‐terminal fragments are present elsewhere within the CNS. We have previously demonstrated truncation of TDP‐43 in the motor cortex of Alzheimer's disease patients by MS‐PRM where classical TDP‐43 pathology was evident by IHC only in the limbic cortex [19].

Of significant interest is that the major domain identified to be abnormally phosphorylated in ALS cases, S409/410, is absent from the Y374X truncated protein [34, 35]. However, intracellular inclusions in CNS tissue from our case label with antibodies to that domain, suggesting that full‐length TDP‐43 is also present in the inclusions. It is known that mutant TDP‐43 can sequester wild‐type TDP‐43 into inclusions in cell models, and this sequestration has also been demonstrated with upregulation of atypical shortened isoforms [36]. The neuropathological findings from this case provide direct evidence of this process occurring in vivo.

In conclusion we have demonstrated that the Y374X TDP‐43 truncating mutation is very likely to be a cause of autosomal dominant ALS. Moreover this mutation is associated with typical TDP‐43 pathology at a macromolecular scale in the spinal cord but also with atypical insoluble TDP‐43 protein fragments and reduced TDP‐43 protein function. These data extend the spectrum of observations associated with TDP‐43 pathology in ALS and will inform future work aimed at ameliorating neurotoxicity associated with this protein.

## AUTHOR CONTRIBUTIONS

J.C.K. C.H., P.J.S, R.J.M, J.R.H, and J.K. conceived and designed the study. J.C.K., E.F., J.R.H, M.S., S.P.A., T.M., T.S., L.C., G.M.H., C.H., J.K., and S.B.W, were responsible for data acquisition and data analysis. J.C.K., E.F., M.R.T., K.T., O.A., J.R.H, T.H.J., M.S., T.M., S.P.A., T.S., L.C., G.M.H., C.H., J.K. S.B.W, and P.J.S. were responsible for the interpretation of the findings. T.H.J., J.C.K., E.F., M.S., C.H., J.R.H., R.J.M., and P.J.S. drafted the manuscript with assistance from all authors. All authors meet the four ICMJE authorship criteria, and were responsible for revising the manuscript, approving the final version for publication, and for accuracy and integrity of the work.

## CONFLICT OF INTEREST

Emily Feneberg, Kevin Talbot, Martin R. Turner, and Olaf Ansorge have a patent filed for the TDP‐43 sequences described in this work (Patent application no. 2004863.3). Other authors delare no conflict of interest.

## Supporting information


**Table S1** Sequenced genes with no identified ALS‐associated mutation in members of the Y374X‐TDP‐43 ALS pedigree.

## Data Availability

The data that support the findings of this study are available from the corresponding author upon reasonable request.
