# Supplementary material for: Atypical TDP‐43 protein expression in an ALS pedigree carrying a p.Y374X truncation mutation in TARDBP
Source: Brain Pathol. 2022 Jul 24;33(1):e13104. doi: 10.1111/bpa.13104 (PMC9836368; doi:10.1111/bpa.13104)
Supplement: Supplementary file 1 — Table S1 Sequenced genes with no identified ALS‐associated mutation in members of the Y374X‐TDP‐43 ALS pedigree. [file BPA-33-e13104-s001.pdf]

Supplementary Table 1: Sequenced genes with no identified ALS-associated mutation in members of the Y374X-TDP-43 ALS pedigree

C9ORF72
ALS2
ANG
ANXA11
ARHGEF28
ATXN2
CHCHD10
CHMP2B
CYP27A1
DAO
DCTN1
ERBB4
EWSR1
FIG4
FUS
GBA1
GRN
HNRNPA1
HNRNPA2B1
MAPT
MATR3
NEFH
NEK1
OPTN
PFN1
PRPH
SETX
SIGMAR1
SOD1
SPAST
SPG11
SPG20
SQSTM1
SS18L1
TAF15
TBK1
TUBA41
UBQLN2
VAPB
VCP
VPS54
VRK1
